# Numerical analysis of the relationship between mixing regime, nutrient status, and climatic variables in Lake Biwa

**DOI:** 10.1038/s41598-022-22124-0

**Published:** 2022-11-16

**Authors:** Jinxin Zhou, Takero Yoshida, Daisuke Kitazawa

**Affiliations:** 1 Institute of Industrial Science, The University of Tokyo, 5-1-5 Kashiwanoha, Kashiwa, Chiba 277-8574 Japan; 2 Department of Ocean Sciences, Tokyo University of Marine Science and Technology, 4-5-7 Konan, Minato-ku, Tokyo, 108-8477 Japan

**Keywords:** Climate-change ecology, Ecological modelling, Freshwater ecology, Hydrology, Limnology, Climate-change impacts

## Abstract

As awareness of climate-related freshwater quality problems increases, more research is needed to better understand how climate impacts water quality. Climate has significant impacts on the mixing regime and nutrient status of lakes. However, the relationship between climatic variables, mixing regime, and nutrient status in large monomictic lakes requires further study. Here we applied a three-dimensional ecosystem model to the large monomictic Lake Biwa, where hypoxia had recurred over the past 60 years. The model was validated using monitoring data, and the relationships among mixing regime, nutrient status, and climatic variables have been investigated. The turnover timing, which represented the mixing regime, varied by 36 days and depended most on wind speed but least on air temperature. In the early period prior to waste-water treatment there was a strong linear relationship between phosphorus and deep-water dissolved oxygen (DO) concentrations. Following this period, the relationship weakened but remained significant. In addition, we find a weak relationship between turnover timing and deep-water DO concentrations. We suggest that later turnover timing may favor lower deep-water DO concentrations, which in turn may favor release of legacy nutrients from sediments. Although waste-water treatment has improved conditions in the lake, climate change induced alteration of turnover timing may adversely influence water quality. Maintaining water quality under continued warming may require more rigorous controls on phosphorus loading to the lake.

## Introduction

An equitable, stable, and sustainable society is impossible without a guaranteed supply of high-quality freshwater. However, access to high-quality freshwater is still a concerning challenge as a direct (e.g., eutrophication) and indirect (e.g., climate change) result of human activities^1^. Although freshwater ecosystems confront comparable stressors, they respond differently on sub-seasonal to multi-decadal timescales. Eutrophication in rivers and lakes has received widespread recognition and has extensive management strategies^2,3^. To date, eutrophication in rivers remains one of the world’s most pressing concerns^4,5^. However, eutrophication in lakes has been effectively controlled^6^, and some lakes are now experiencing oligotrophication (the reversal of the eutrophication process) as a result of wastewater treatment^7^. On the other hand, climate-driven problems with water quality remain poorly understood and lack appropriate management strategies due to complexly interlinked processes^8,9^. For example, the complex shifts in thermal regimes due to global warming have drawn scientific attention, especially in lakes^8^.

Research on the impacts of anthropogenic activities on lakes has been extensive. From 1963 to 1967, Lake Washington recovered from heavy eutrophication after diverting the sewage effluents^2^. Similar practices have shown a relationship between eutrophication and nutrient enrichments in lakes^9^. Phosphorus, whose global circulation has almost tripled compared to its natural mobilization, has been identified as the common driver of eutrophication along with nitrogen^10^. More recently, a meta-analysis indicates that input concentrations, whether dissolved or particulate, always have a proportionate impact on lake phosphorus concentrations^6^. On the other hand, research on lake responses to climate change is still ongoing. Studies in this field similarly started from a single lake, albeit over a longer time frame, like the research on Lake Washington from 1964 to 1998^11^. Then studies were expanded to a region, like the major six lakes in Eastern Asia^12^, the representative 17 lakes in Europe^13^, or the 142 lakes in Wisconsin, USA^14^. Climate-driven shifts in the lakes’ surface water temperature were initially investigated on a global scale^15^, followed by changes in lake thermal regimes in 139 lakes worldwide^8^. A state-of-the-art lake model for 635 lakes worldwide^16^ and an ensemble of process-based models across the Northern Hemisphere^17^ showed that the above changes in thermal structures are primarily affected by the lakes’ hydrodynamics, particularly the lakes’ mixing regime. The conclusion agrees with the observation of continuous monitoring data (3-hourly and hourly) in Lake Michigan over a 30-year period^18^. However, long-term shifts in lake ecosystem responses, including the link between mixing regime and nutrient status, have not been generalized globally or regionally. Our understanding of mixing regime shifts and their associations to nutrient state will be improved by well-designed local studies^19–22^.

**Figure 1:**
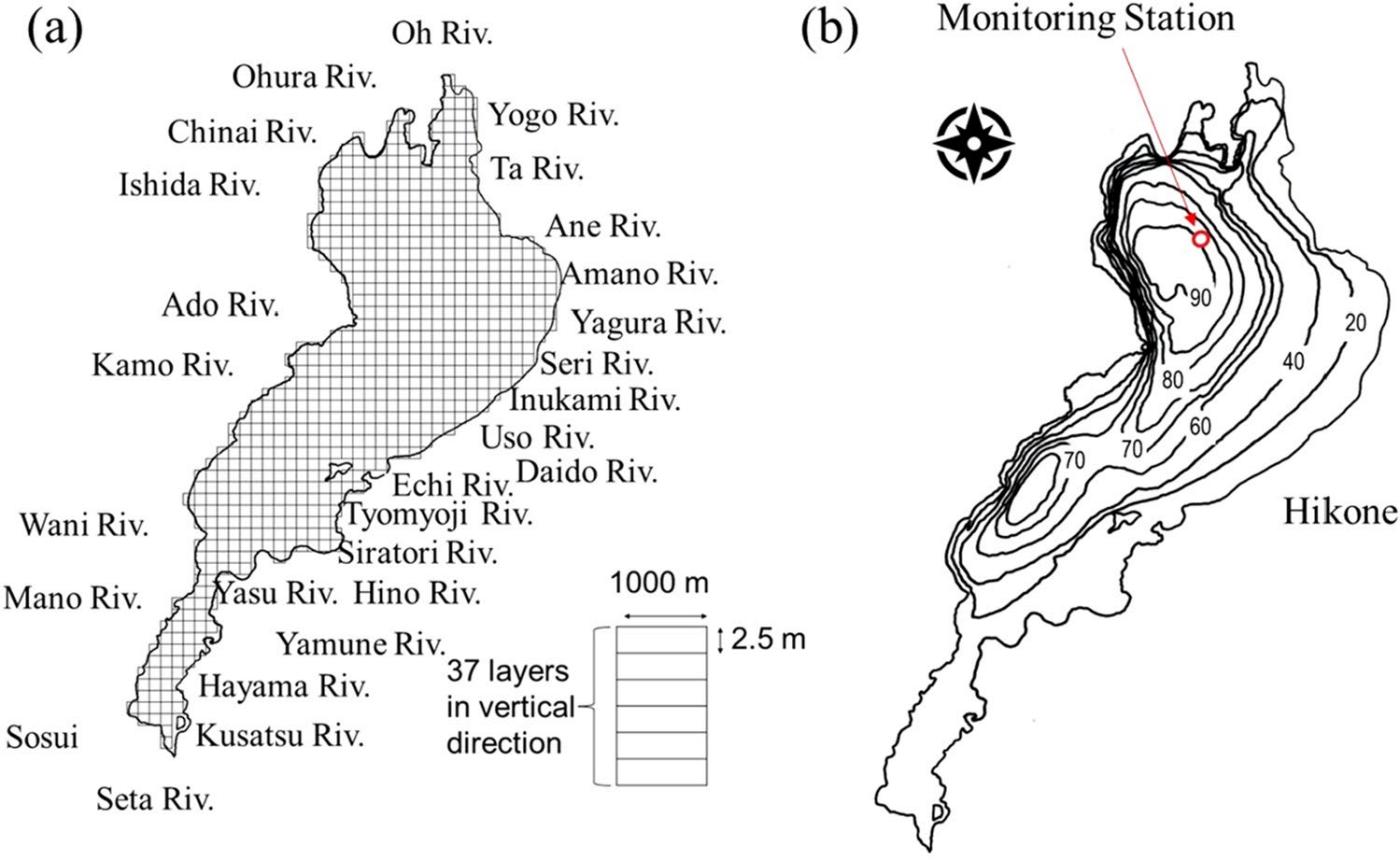
The map and computational conditions of Lake Biwa. Computational conditions for (**a**) the grid system and the 25 nearby rivers, (**b**) one station for water quality monitoring, and one station (Hikone) for meteorological observation.

Lake Biwa, the largest lake in Japan and one of the ancient lakes in the world, is an iconic lake typically valued for its drinking waters (and see more details in “Methods” section; Fig. 1). Lake Biwa has experienced multiple environmental problems over the last 60 years^23^. Due to the high external inputs of nitrogen and phosphorus, anthropogenic eutrophication with concomitant phytoplankton proliferation and hypoxia has been identified in the littoral and offshore areas since the 1960s. Eutrophication ceased in the 1980s, shortly after the establishment of wastewater treatment. Water quality has improved since the 1990s based on the amount of organic matter in the hypolimnion^23^. Recent changes in water quality appear to be related to climate change^12,24^. Atmospheric temperature and wind speed are the two basic meteorological variables, with wind speed having the greatest impact on the stratification and oxygen transport in Lake Biwa^25^. When the demand for dissolved oxygen exceeds its supply, hypoxia eventually results, especially at the bottom where oxygen transported from the surface is the major source of supply^12^. Such ecosystem responses to climate change, including hydrodynamic and biogeochemical processes, have long been numerically studied, as in Lake Erie^26^. A three-dimensional ecosystem model was adopted in the previous study that successfully disentangled the effects of climate change on the ecosystem in Lake Biwa from 1955 to 2005^24^. However, a holistic understanding is essential because warming and hypoxia continue in Lake Biwa. Understanding the progression of ecosystem responses to warming in Lake Biwa can help improve the understanding of mixing regime shifts in large monomictic lakes worldwide.

We expanded the previous numerical study to reproduce the ecosystem dynamics from 1955 to 2018 in Lake Biwa. Based on the simulation results, this study aims to first elucidate how the mixing regime and nutrient status varied over time. The beginning date of full water mixing (turnover), which describes the lake’s water turning over from top to bottom, was used as the criterion for evaluating the mixing regime. Two dominant contributors to eutrophication, nitrogen and phosphorus, were the focus of nutrient status. The interlinked relationships between the mixing regime, climate conditions, and nutrient status were then disentangled. Finally, the ramifications of the above shifts were discussed on the bottom concentration of dissolved oxygen (DO), a crucial index of water quality. Based on the effects, recommendations for safeguarding water quality were provided.

## Results

### Model validation

The annual and seasonal variations in water temperature and DO concentrations were successfully reproduced by the model (Supplementary Fig. S1). The lake’s water temperature was validated from April 10, 1978, to March 19, 2018. A very good model accuracy between observed and modeled state variables has been obtained at the surface with a root mean square error (RMSE) of 1.47 °C and a normalized root mean squared error (NRMSE) of 0.09 (Supplementary Fig. S2a). A slight discrepancy in bottom water temperature was observed before 1995 (Supplementary Fig. S1a); however, this discrepancy did not affect the overall reproducibility, as seen by the RMSE of 0.90 °C and NRMSE of 0.12 (Supplementary Fig. S2b). The lake’s DO concentrations were validated at the bottom between February 1, 1978, and February 18, 2010. With an RMSE of 1.76 mg/L and an NRMSE of 0.27 (Supplementary Fig. S2c), it is slightly less accurate than water temperature (with NRMSE of 0.12 at the bottom). There were noticeable discrepancies around 1986 when the observed concentrations were far below the model output (Supplementary Fig. S1b). However, this discrepancy only became apparent in a few simulated months.

### Mixing regime

**Figure 2:**
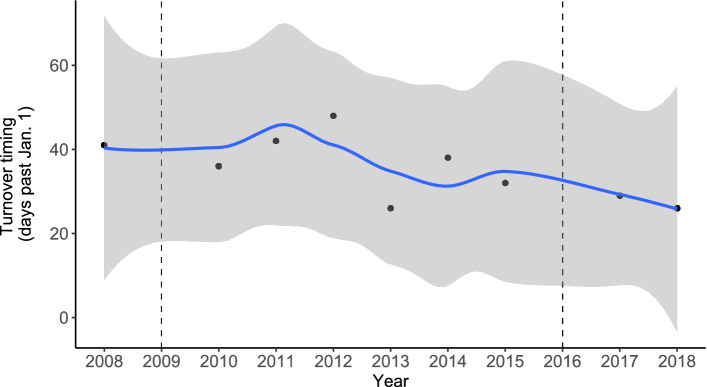
The simulated time variations in the timing of turnover in Lake Biwa from 2008 to 2018. Dots show the value of turnover timing, and dashed lines represent the failure of turnover. A fitting curve (the blue line) exhibits general change trends with a 95% confidence interval.

Due to the high simulation accuracy in recent years, we checked the frequency of turnovers from 2007 to 2018. The full water mixing (turnover; see an example in Supplementary Fig. S3) was determined by the curve shape of water temperature versus DO concentration (Supplementary Fig. S4). The value of water temperature and DO concentrations at the bottom was used to plot the above curve because the renewal of deep water is an iconic feature of turnover. A turnover appears when there is a turning point at the curve, like that in 2008, rather than a relatively straight line, like that in 2009. Based on this criterion, 2007, 2009, and 2016 were the only three years without a turnover. The water temperature and DO concentrations in the above three years varied significantly in March; however, successful turnovers were accompanied by early water mixing (Supplementary Fig. S4). We used the beginning date of turnovers (days past January 1) to illustrate turnover timing (Fig. 2). Although a turnover theoretically means an identical state of the surface and bottom water, the turnover timing was numerically determined by the difference in water temperature of 0.5 °C and DO concentration of 0.1 mg/L between the surface and bottom given the numerical accuracy (Table 1). When the simulation and observation dates were compared, a very good agreement has been reached with a median difference of four days (Table 1).

**Table 1:** Comparison of the simulation results with the observational data regarding the timing of turnover.

Year	Observational data	Simulation results	Difference (days)
2007	March 19	Failure	–
2008	February 12	February 11	1
2009	February 23	Failure	–
2010	February 8	February 6	2
2011	January 24	February 12	19
2012	February 13	February 18	5
2013	January 29	January 27	2
2014	February 17	February 8	9
2015	February 2	February 2	0
2016	March 14	Failure	–
2017	January 26	January 30	4
2018	January 22	January 27	5

### Nutrient status

We selected nitrogen and phosphorus, represented by the dissolved inorganic forms of nitrogen (DIN) and phosphorus (DIP), to study the nutrient status in Lake Biwa using simulation results, and investigated how their time-series fluctuations varied across all depths during the simulation period (Supplementary Fig. S5). First, the subsurface nutrient concentrations were higher than those at the surface. However, compared to DIN concentrations, the difference in DIP concentrations between the surface and subsurface was far greater. Second, although nutrients exhibited seasonal variations, their concentrations have notably increased since 1975. More recently, the annual DIN concentrations appeared to remain consistent, while the annual DIP concentrations appeared to fluctuate.

**Figure 3:**
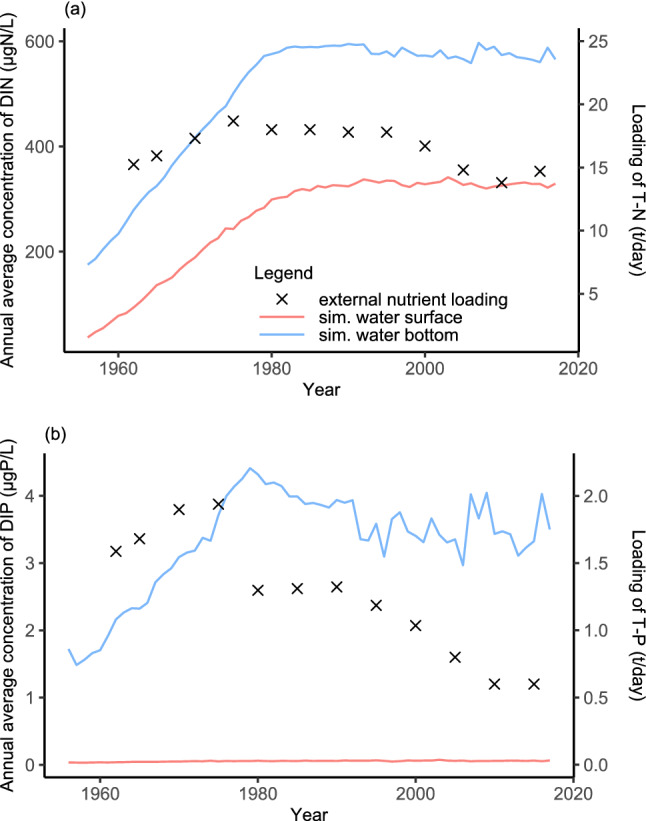
The fluctuation of lake nutrient concentrations at the surface (red lines) and bottom (blue lines) and external nutrient loadings (points): (**a**) nitrogen and (**b**) phosphorus.

The annual average concentrations at the surface and bottom were calculated to quantitatively compare nutrient concentrations at different water depths and simulation times (Fig. 3). Nitrogen concentrations have almost been identical since 1980, although the gradient is slightly different. Around 1977, the bottom DIN concentrations reached their steady stage whereas surface concentrations became stable around 1980. The bottom DIP concentrations peaked in 1977 and decreased for several years. Although the recent value oscillated, the average value appeared to be stable. The surface DIP concentrations exhibited a different pattern while remaining at zero over the simulation period. On the other hand, the effects of external nutrient loading have been examined. Because there are uncertainties in sewage treatment and surface runoff, only the estimates published by the Shiga Prefectural Government were adopted here (Fig. 3). External loading for the two nutrients peaked around 1975 and significantly decreased between 1980 and 2000. The annual average DIP concentrations, however, varied but did not decrease proportionately with the decline in external TP loading. In particular, the external TP loading was 50% lower in 2010 compared to 2000, while a slight change was observed in DIP concentrations.

## Discussion

### Model validation

Based on the time-series validations of water temperature and DO concentration, model accuracy improved gradually, despite several discrepancies at the beginning of the simulation (Supplementary Fig. S1). The model is primarily driven by a set of boundary data, including wind speed, solar radiation, and precipitation data^24,25^. From this perspective, more high-quality boundary data promotes better numerical reproducibility. However, meteorological data collection was challenging due to the early observation equipment limitations and low observational accuracy compared to current data. The temporal inconsistency of accuracy in observational data has been eliminated to a large extent by fitting a regression curve^24^. Spatial resolution is the other issue. Possessing spatially constant values for all boundary conditions complicates the numerical reproducibility of variations on finer scales.

The relationship between turnovers and the curve shape of water temperature versus DO concentration is theoretically sound^27,28^. In the last stage of stratification in the lake, water temperature and DO concentration near the bottom are more likely to slightly increase due to thermal diffusion and DO supplies from the upper water. If a turnover occurs, the whole column of water is mixed strongly (Supplementary Fig. S3). Bottom water temperature decreases due to surface water cooling, and DO concentration increases, due to surface water replenishment and increased oxygen solubility. If the turnover fails, only the partial column of water is mixed, causing a delay in the timing of deep-water renewal (Supplementary Fig. S3). However, the upper water in later months, like that in March, has been rapidly warmed, resulting in an increase in the bottom water temperature. For example, in 2007 and 2016, the simulated water temperature and DO concentration fluctuated within a limited range in February and then skyrocketed in March, after mixing with the warmed surface water (blue points in Supplementary Fig. S4). On the other hand, explicit definitions of turnover timing are challenging. The threshold used to judge turnover timing is reliable because the results matched the observation. The turnover timing varied by 36 days in Lake Biwa during the simulation period, which is comparable to that observed in other lakes, such as approximately 21 days in Heiligensee, Germany over a 17-year timespan^29^, 16 days in Lake Washington over a 40-year timespan^30^, and 28 days in Blelham Tarn over a 41-year timespan^31^.

### Variables affecting the mixing regime

Determining variables that affect the mixing regime is essential to improve understanding and enable future projections^16–18^. Air temperature, wind speed, cloud cover, precipitation, water density, and lake transparency are all potential variables. We, here, compared the above variables to the turnover timing in Lake Biwa. The meteorological inputs in this study provided data for air temperature, wind speed, cloud cover, and precipitation. Water density and particulate organic carbon (POC) concentration representing lake transparency were the model’s outputs. The annual averages and cold season (November–April) values of the above variables were calculated over the simulation period (Supplementary Fig. S6). Annual averages illustrate general long-term warming trends^18^, while cold season values particularly determine the timing of turnover^17^. However, in Lake Biwa, air temperature during the cold season fluctuated greatly compared to the annual averages. A random forest analysis^17^ has been conducted between the turnover timing and the above two variable sets (cold season values versus annual averages) in Lake Biwa, and the cold season values better explained the turnover timing (35.39% versus 18.48%). The results agree with the conclusion drawn from the previous sensitivity tests, which indicated the relative importance of air temperature and solar radiation during winter based on 40 scenarios^32^.

**Figure 4:**
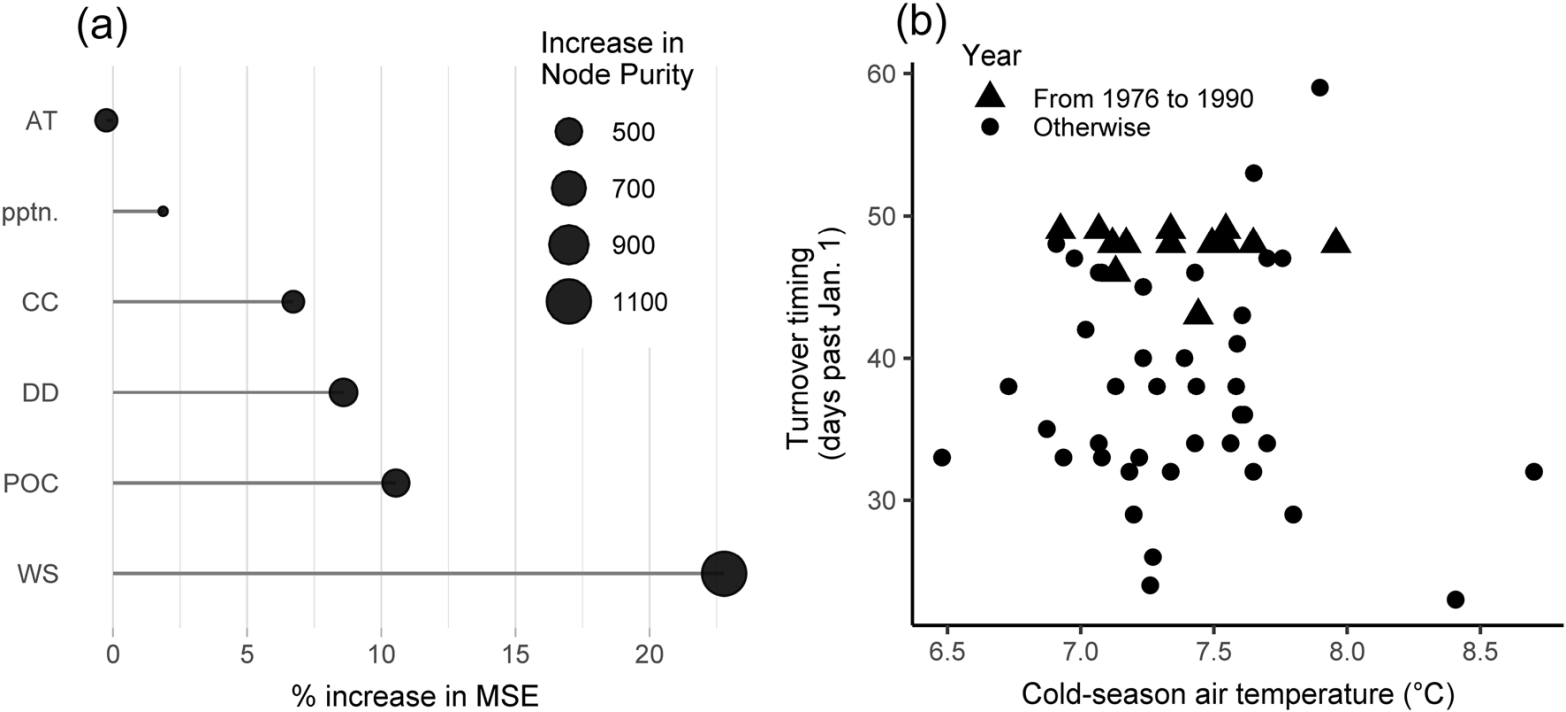
Analysis results of the relationship between potential variables and turnover timing: (**a**) the importance of variables importance using a random forest analysis, and (**b**) the relationship between the cold season air temperature and the timing of turnover. Variable importance is calculated using the percentage increase in mean square error (MSE) and the increase in node purity. Higher values illustrate the greater importance of the variable. Variables include air temperature (AT), precipitation (pptn.), cloud cover (CC), the difference in density (DD), POC, and wind speed (WS).

The importance of variables was estimated based on the random forest analysis using the cold season data (Fig. 4a). Wind speed dominates the timing of turnover, which is consistent with the previous studies^17,25^. The POC concentration, the difference in water density between the surface and bottom, and cloud cover have moderate effects on the timing of turnover. However, air temperature is less important, which is contrary to the turnover mechanism^17,24,32^. A re-confirmation was conducted of the relationship between turnover timing and air temperature (Fig. 4b and Supplementary Fig. S7). The cool air generally encourages an early turnover, albeit with several anomalies. The turnover timing between 1976 and 1990 remained constant independent of climate change, and the period coincidently had a substantial nutrient fluctuation (Fig. 3). As a result, it is essential to investigate the nutrient status further.

### Lake nutrient concentrations

Because phosphorus is the limiting nutrient in Lake Biwa and DIP concentrations can be effectively limited by regulating external loadings as practiced (Fig. 3), DIP concentrations become the focus of this discussion for nutrient status. However, the DIP concentrations disproportionately responded to the external loadings of total phosphorus (TP) in Lake Biwa. Although external TP loading itself fails to determine lake phosphorus concentrations due to the hydrodynamics of lakes^33^, Lake Biwa exhibited insignificant changes in the inflow rate or the retention time (and see an example of the surface flow in Supplementary Fig. S8). Therefore, it can be assumed that the hydraulic loading remained constant, and the input nutrient concentrations were proportionate to the external nutrient loadings in Lake Biwa. This finding contradicts a recent meta-analysis that highlighted a deterministic relationship between input nutrient concentrations and lake nutrient concentrations, based on steady-state mass balance models^6^. The possible reason is the dynamics of the lake’s ecosystem^22^, which have been considered in this study. For example, the surface DIP concentrations were almost nonexistent regardless of the external TP loadings in Lake Biwa, supporting that phosphorus is the limiting nutrient in Lake Biwa^34,35^. The low DIP concentrations at the surface may be caused by the rapid recycling of phosphorus because the amount of phosphorus available for phytoplankton is easily affected by the feedback mechanism between phytoplankton photosynthesis and the phosphorus released from the water^35,36^.

### Hypoxia and strategies

The variations in DO concentration are the public’s top concern as it relates to hypoxia, a key indicator of water quality. Lake bottom, among all water depths, is more sensitive to small changes in oxygen conditions^12^. In Lake Biwa, the annual minimum DO concentrations ranged from 2 to 5.5 mg/L over the last 60 years (Supplementary Fig. S9). The decrease in DO concentrations in the early period, typically till the 1980s, was mainly caused by nutrient enrichments (Fig. 3). The nutrient enrichment-induced heavy eutrophication eventually accelerates the rate of DO depletion^2^. After eutrophication was controlled in the 1980s, climate change became the dominant stressor^23^. There remains much uncertainty surrounding the relationship between climatic variables-related turnover timing and hypoxia in Lake Biwa^12^. We, therefore, first investigate the relationship between hypoxia and turnover timing, and then concentrate on nutrients to alleviate hypoxia.

**Figure 5:**
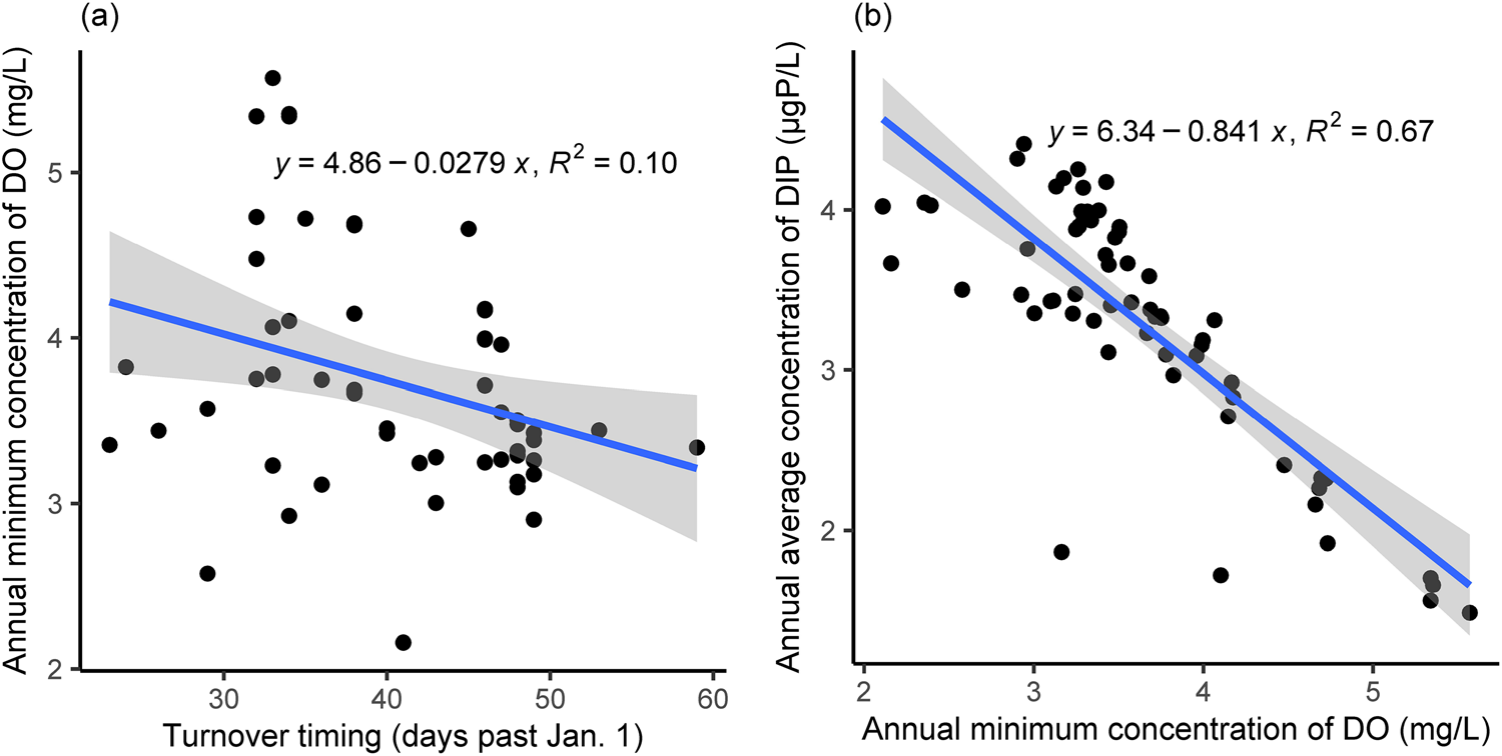
The linear regression results of the relationship: (**a**) between turnover timing and annual minimum concentration of DO, (**b**) between the annual minimum concentration of DO and annual average concentration of DIP. The simulation results at the monitoring station were used for analysis.

Although the relationship between turnover timing and DO concentrations is quite weak (*R*^2^ = 0.10), there is a general decrease in DO concentrations with increasing turnover timing (Fig. 5a). On the other hand, a linear relationship has been found between DIP concentrations and DO concentrations, with an *R*^2^ of 0.67 (Fig. 5b). The slope of –0.841 μgP/mgDO means an increase in DIP concentrations by approximately 0.841 μgP/L causes a decrease in DO concentrations by 1 mg/L. Note that the simulation results were compared over the whole period, and eutrophication-induced hypoxia differs theoretically from climate-induced hypoxia. Additional testing has been conducted to distinguish the effects of two stressors (eutrophication- and climate-induced hypoxia; Supplementary Fig. S10). Before 1980 when eutrophication progressed, the annual minimum DO concentrations and the DIP concentrations had a stronger linear relationship (*R*^2^ = 0.89). Although waste-water treatment has improved conditions in the lake, climate change induced alteration of turnover timing may adversely influence water quality. However, the relationship weakened dramatically with an *R*^2^ of 0.10 after 1980, when climate change dominated hypoxia. The lower *R*^2^ value indicates that climate-related hypoxia is more complex as concluded previously^37,38^. The two possibilities are as follows. First, there can be a legacy of hypoxia related to eutrophication. The DO recovery at the bottom of Lake Biwa was complicated by the low DO concentration in 1980 and the delayed timing of turnover; similar phenomena have been observed in the Lake of Zurich^22^. Second, ecosystem dynamics could help explain the difficulty in predicting hypoxia at the bottom. Phytoplankton fully exploits phosphorus at the surface, as explained above, then the death and sinking of the surface phytoplankton are accompanied by the sedimentation of phosphorus to the bottom as modeled. Bacteria break down the sinking phytoplankton, releasing phosphorus and consuming DO in the process. Additional DO consumption lowers the bottom DO concentration, which in turn encourages phosphorus release from the sediment in a low DO environment^22^. Such unfavorable feedback between DIP and DO concentrations are strengthened by prolonged stratification and eventually accelerates the development of hypoxia. However, future research is necessary because this numerical model simplified the relationship between water and sediment. The sinking of organic carbon into sediment is integrated in the model, and due to the decomposition of organic carbon in the sediment, nutrients are released into and oxygen is depleted in the water. Despite that, the trends between DO and DIP concentrations stay the same under climate change (Fig. 5b), and thus controlling lake phosphorus is beneficial to the Lake Biwa hypoxia.

## Methods

### Numerical model

The three-dimensional ecosystem model consists of two interlinked components—a physical submodel and a pelagic ecosystem submodel. The governing equations of the physical submodel, under hydrostatic and Boussinesq approximations, consist of the momentum equation, the continuity equation, the advection and diffusion equations, and the state equation for calculating current velocity, pressure, water temperature, and density^24^.

The model structure regarding the pelagic ecosystem submodel is built by modeling each state variable's biophysical and biochemical activities. State variables include phytoplankton, zooplankton, DIP, DIN, DO, and POC (Supplementary Fig. S11). Take phytoplankton as an example. Photosynthesis contributes to biomass increment while respiration, mortality, and predation cause biomass to decline, with detailed descriptions referring to Zhou^39^.

When integrating the physical and pelagic ecosystem submodels, we assume that all state variables in the pelagic ecosystem are passively transported by water current, and therefore an advection–diffusion equation is applied as follows:

1 $$\frac{\partial C}{\partial t}+\frac{\partial \left(uC\right)}{\partial x}+\frac{\partial \left(vC\right)}{\partial y}+\frac{\partial \left(wC\right)}{\partial z}=\frac{\partial }{\partial x}\left({A}_{H}\frac{\partial C}{\partial x}\right)+\frac{\partial }{\partial y}\left({A}_{H}\frac{\partial C}{\partial y}\right)+\frac{\partial }{\partial z}\left({K}_{H}\frac{\partial C}{\partial z}\right)+{{q}_{C}+R}_{C}$$

where *C* is each state variable, i.e., phytoplankton; ${q}_{C}$ is the variation in each state variable due to biochemical processes; and ${R}_{C}$ is river inputs.

The boundaries include the sidewall, bottom, water surface, and rivers. At the sidewall and the bottom, water current velocity normal to them is set to zero, but velocity parallel to them is considered differently. At the sidewall, free-slip conditions are applied regarding the large mesh size introduced afterward, and at the bottom, frictional stress and kinematic conditions are applied with a constant value. Water temperature and all state variables normal to these two boundaries have a zero-gradient change pattern with no advection or diffusion fluxes of heat across them. Wind stress is set to be uniform across the simulation area at the surface, and the heat flux through the surface is determined by solar radiation, net longwave radiation, sensible heat flux, and latent heat flux. There are no chemical exchanges at the surface, while oxygen is supplied through atmospheric exchange using a constant reaeration coefficient and the difference between the DO at saturation and the DO at the water surface (Supplementary Fig. S12). The effects of rivers follow Eq. (1) in terms of chemical materials, and the effects on current velocity are proportional to the water flow rate.

### Lake Biwa

The largest lake in Japan, Lake Biwa, is in the central region of Honshu Island. Lake Biwa has a north–south length of 63 km and a width of 23 km, with a surface area of 670 km^2^, and a maximum depth of 104 m^23^. The lake is divided into two basins: the larger, deeper northeast basin and the shallower little southwest basin, and the two basins are connected by a wide neck with a minimum width of 1.4 km. The North Basin and South Basin's average depths are 44 m and 3.5 m, respectively. From the surrounding mountains, almost 400 rivers of various sizes feed into Lake Biwa. In the catchment region, the average annual precipitation is roughly 1700 mm, and the average annual air temperature is 14 °C. The lake is regarded as a semitropical lake since it never freezes. Thermocline development is observed only in the North Basin from May to November.

### Computational conditions and validation processes

We used a structured grid system for the case of Lake Biwa (Fig. 1a), and the horizontal and vertical grid sizes are 1000 m and 2.5 m, respectively. Water depth was obtained from the Geospatial Information Authority of Japan, with 37 layers in the vertical direction. For boundary inputs, there are 25 inflow rivers and two outflow canals surrounding Lake Biwa (Fig. 1a), with nutrient inputs available from the observation data by the Ministry of the Environment. The river inflow rate was calibrated using the area of the river and the seasonal variation in precipitation, and the river water temperature was considered the same as the atmospheric temperature. Meteorological data were obtained from the Hikone Station (Fig. 1b), which was accessible via the Japan Meteorological Agency. Wind velocity was multiplied by 1.2 to compensate for the difference in values between land and water.

We calculated the ecosystem dynamics from March 1955 to July 2018, with a model time step of 20 s. The value of parameters was set the same as in the previous works^24^. There is one monitoring station to validate the simulation results (Fig. 1), including lake water temperature (at 0.5 m and 90 m; semimonthly in most cases between April 10, 1978, and March 19, 2018) and DO concentration (at 90 m; monthly in most cases between February 1, 1978, and February 18, 2010), obtained from the Shiga Prefectural Government. Furthermore, the Lake Biwa Environmental Research Institute weekly monitored DO concentration of the bottom and surface water at the station with the greatest depth. The validation data of turnover timing has been independently evaluated using the criteria if the bottom and surface DO concentrations both are within the range of 10–11 mg/L.

## Supplementary Information

Supplementary Information.

## Data Availability

All monitoring data and input data for the numerical model are publicly available. Topographic data are available from the Geospatial Information Authority of Japan (https://www.gsi.go.jp/ENGLISH/). The water quality data of rivers surrounding Lake Biwa are available from the Ministry of the Environment (https://www.env.go.jp/en/). Meteorological data are based on the Hikone Station available from Japan Meteorological Agency (https://www.jma.go.jp/jma/indexe.html). The water quality data of Lake Biwa and the estimated external nutrient loadings to Lake Biwa are available from the Shiga Prefectural Government (https://www.pref.shiga.lg.jp/ippan/kankyoshizen/biwako/). The validation data of turnover timing are available from the Lake Biwa Environmental Research Institute (https://www.lberi.jp/; in Japanese). Simulation results generated in the present study are available from the corresponding author upon reasonable request.
