# Supplementary material for: Numerical analysis of the relationship between mixing regime, nutrient status, and climatic variables in Lake Biwa
Source: Sci Rep. 2022 Nov 16;12:19691. doi: 10.1038/s41598-022-22124-0 (PMC9668906; doi:10.1038/s41598-022-22124-0)
Supplement: Supplementary file 1 — Supplementary Information. [file 41598_2022_22124_MOESM1_ESM.pdf]

Supplementary Information

Numerical analysis of the relationship between mixing regime, nutrient status, and climatic variables in Lake Biwa

Jinxin Zhou[1, *], Takero Yoshida[2], and Daisuke Kitazawa[1]

[1] Institute of Industrial Science, The University of Tokyo, 5-1-5 Kashiwanoha, Kashiwa, Chiba 277-8574, Japan.

[2] Department of Ocean Sciences, Tokyo University of Marine Science and Technology, 4-5-7 Konan, Minato-ku, Tokyo 108-8477, Japan.

[*]Corresponding author: Jinxin Zhou
E-mail: jxzhou@iis.u-tokyo.ac.jp.
Institute of Industrial Science, The University of Tokyo, 5-1-5 Kashiwanoha, Kashiwa, Chiba 277-8574, Japan.

Supplementary Figure S1

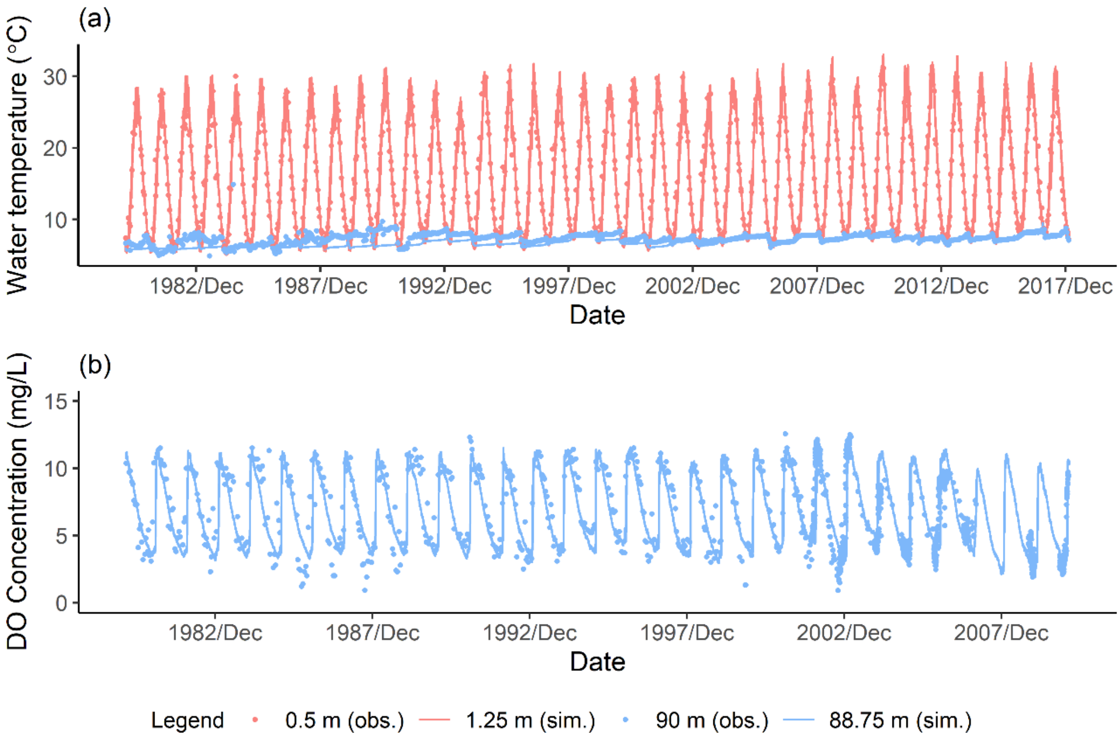

Figure S1. The comparison results between simulation (lines) and observation (dots) at the surface (red) and bottom (blue) for (a) water temperature and (b) DO concentration.

Supplementary Figure S2

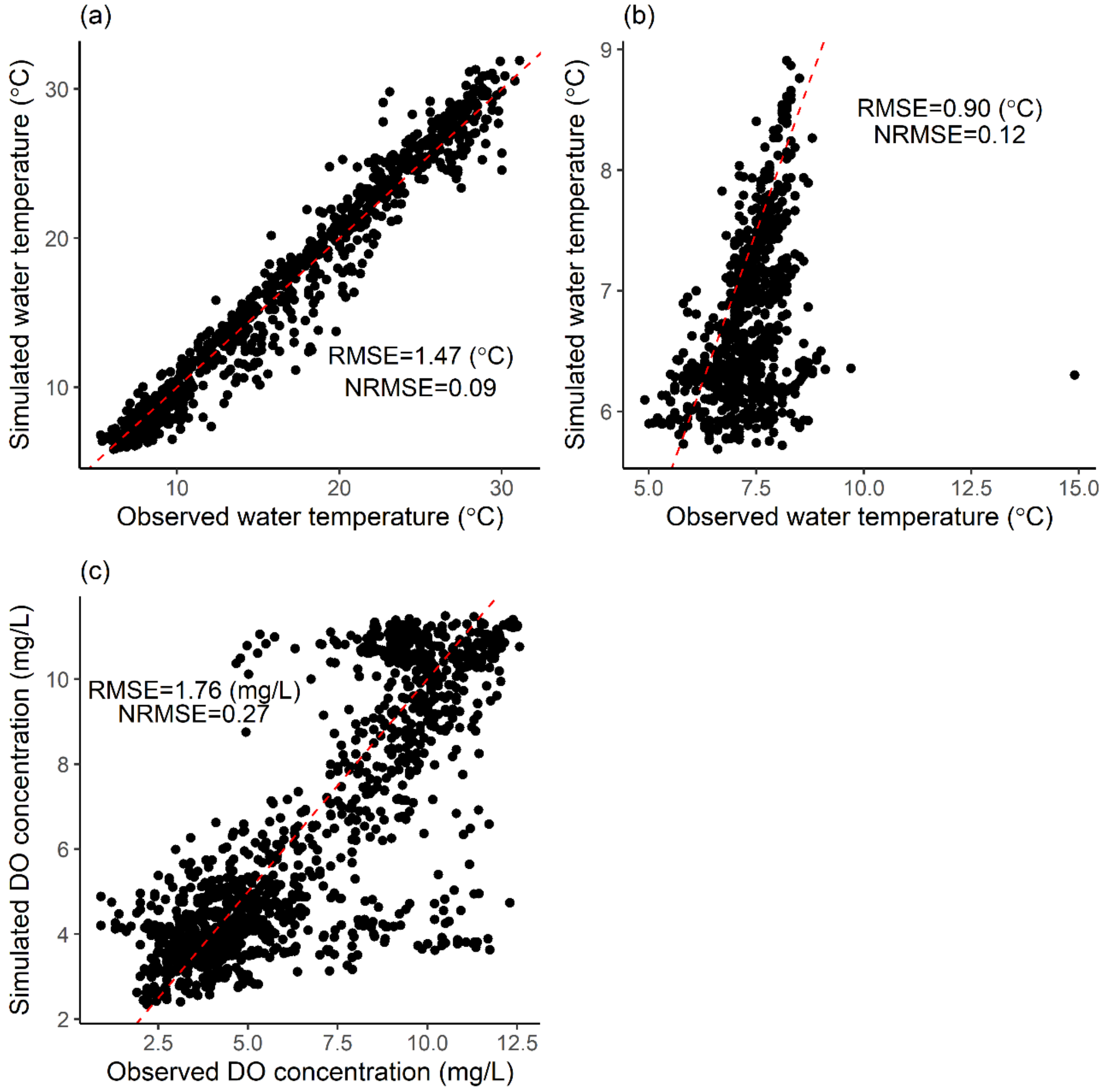

Figure S2. The comparison results between simulation and observation for (a) the surface water temperature, (b) the bottom water temperature, and (c) the bottom DO concentration. The values of RMSE and NRMSE are used to quantitatively evaluate the difference, with red dashed lines representing the ideal situation.

Supplementary Figure S3

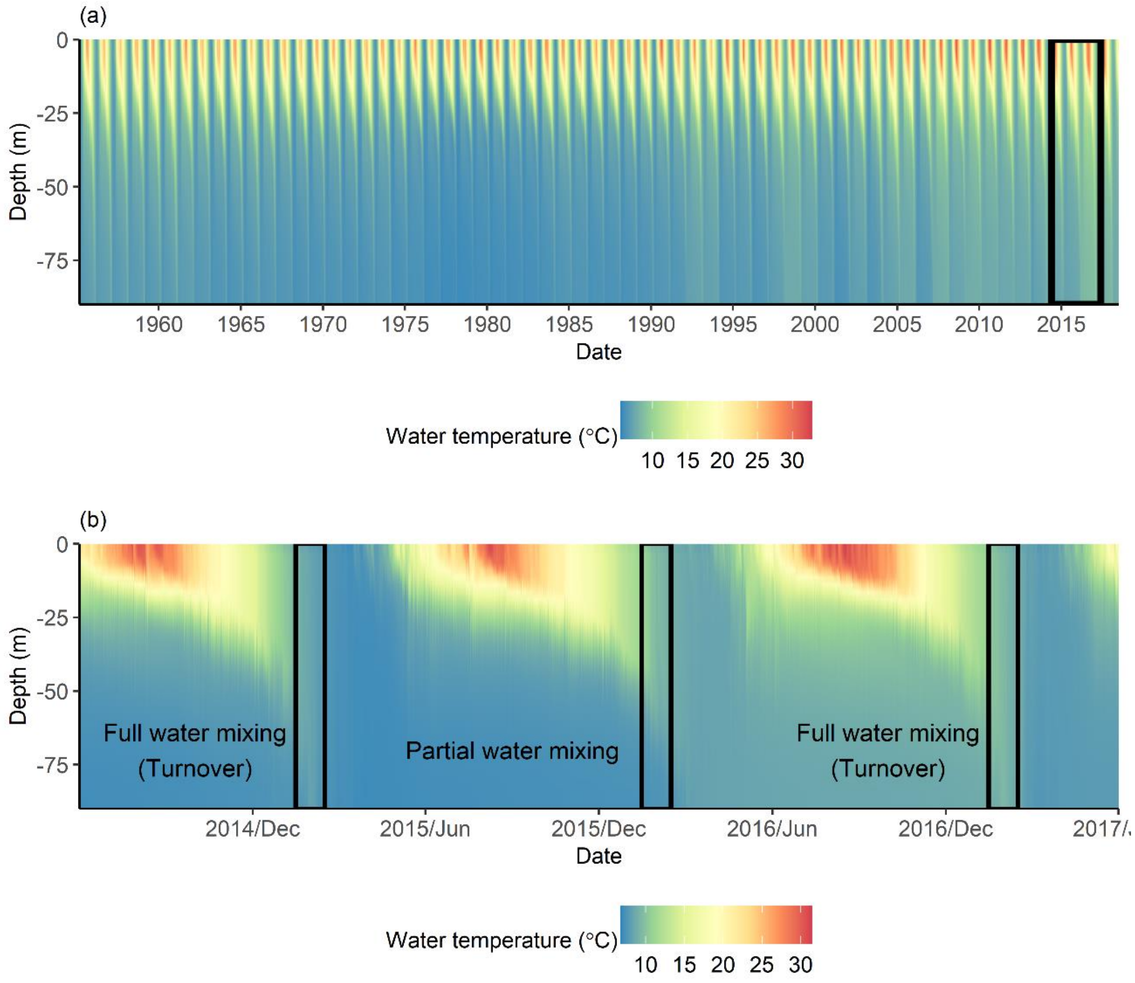

Figure S3. The time-series variations at all depths in water temperature: (a) over the whole simulation period, and (b) from 2014 to 2017 to exemplify the full and partial water mixing. The simulation results at the monitoring station were used for analysis.

Supplementary Figure S4

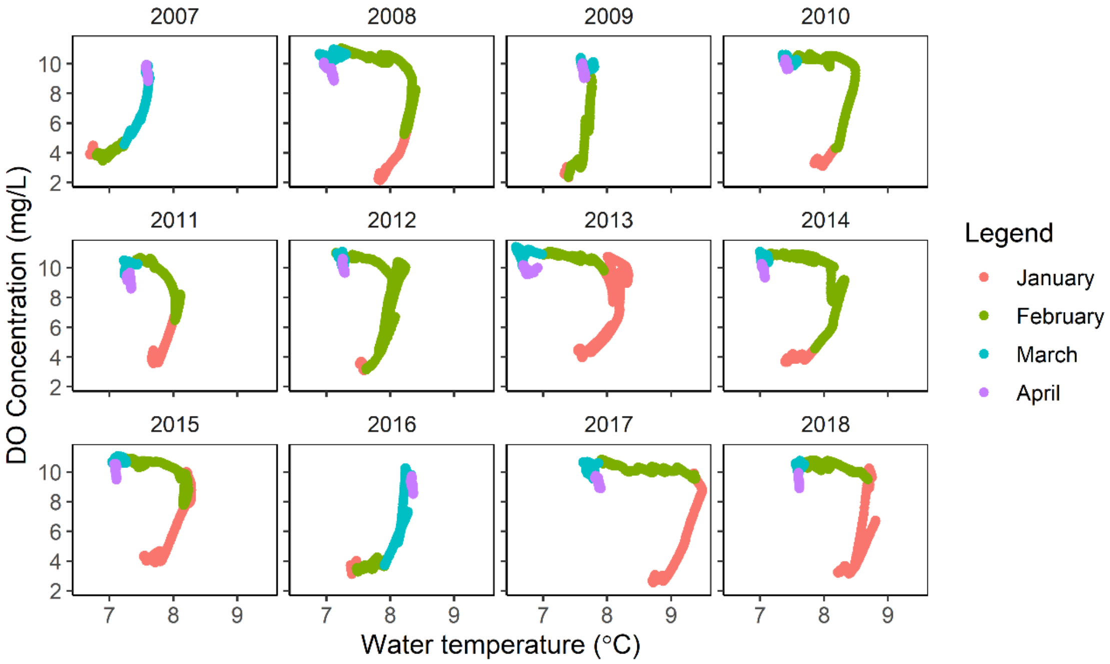

Figure S4. The simulation results of turnover timing from 2007 to 2018. Colors represent months from January to April. The bottom water temperature and DO concentration at the monitoring station were used for analysis.

Supplementary Figure S5

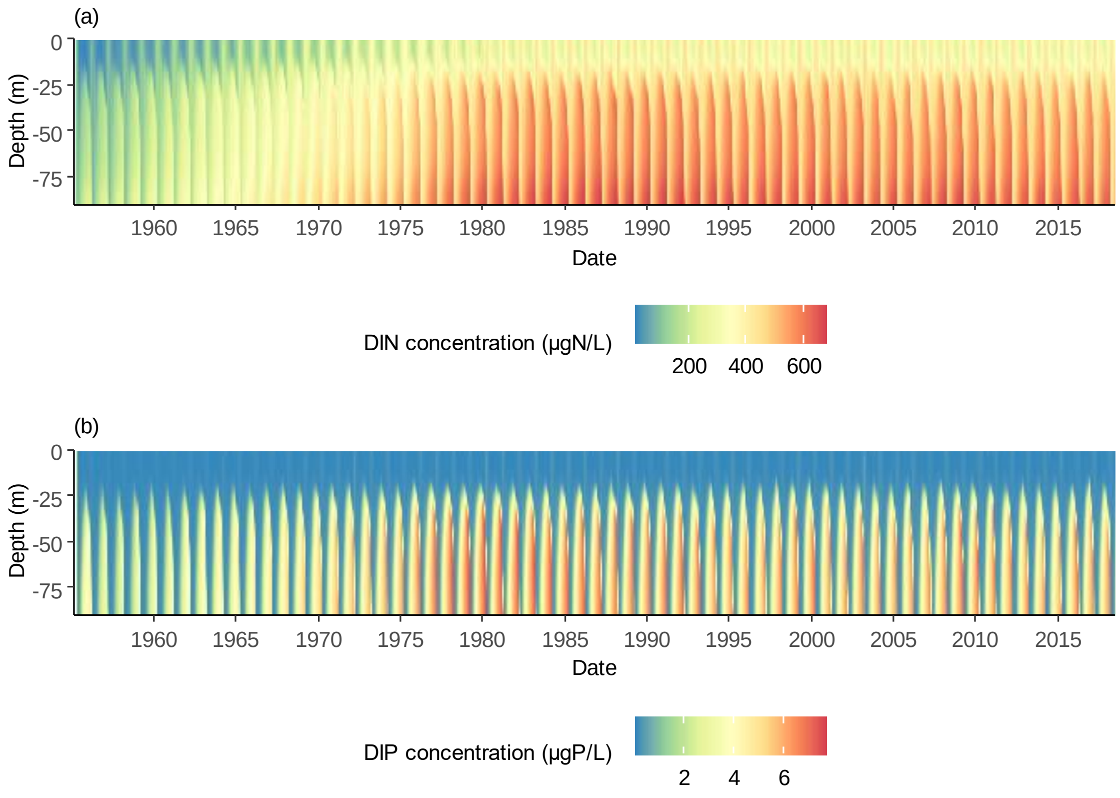

Figure S5. The time-series variations at all depths over the simulation period in the concentrations of (a) DIN and (b) DIP. The simulation results at the monitoring station were used for analysis.

Supplementary Figure S6

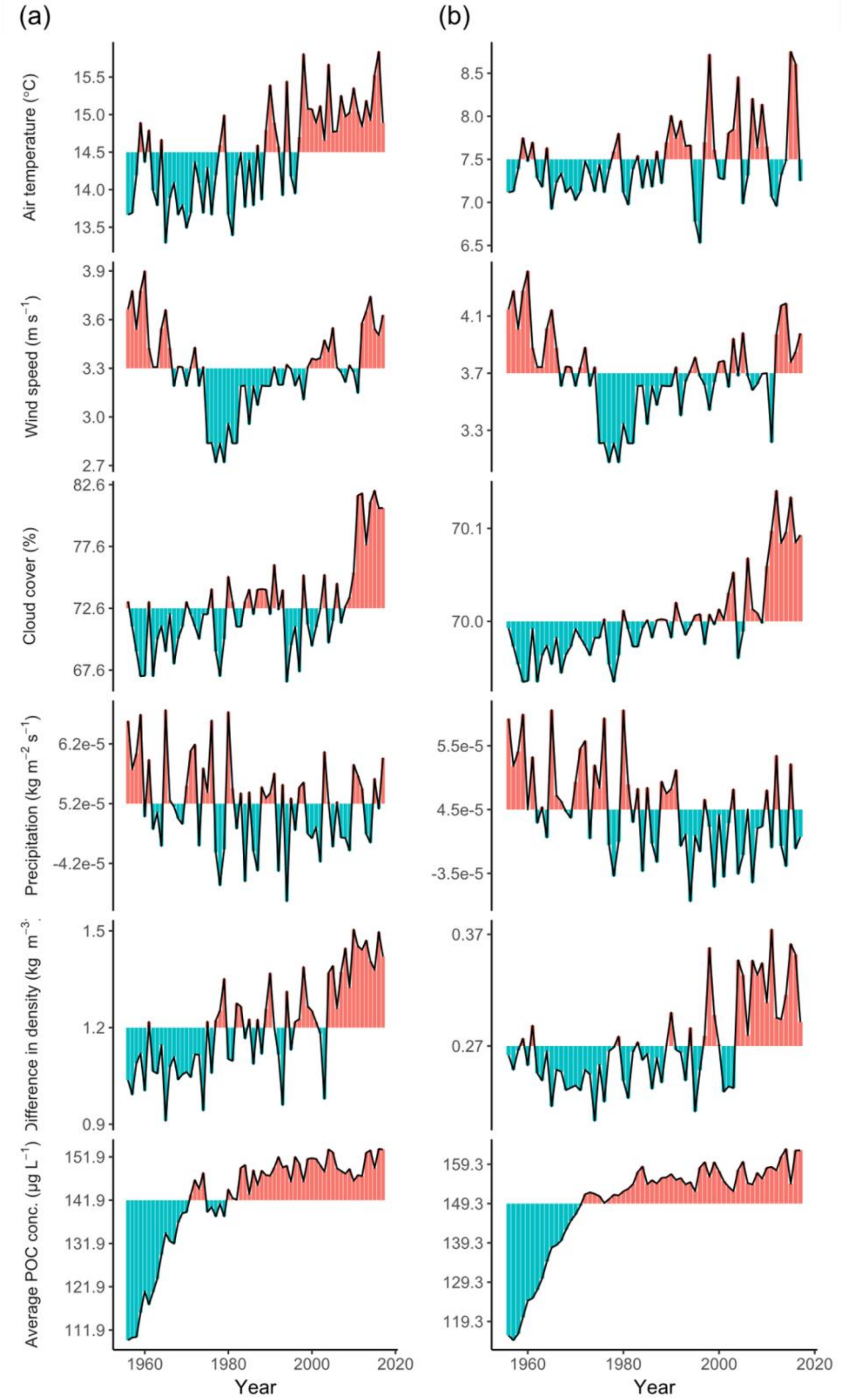

Figure S6. The time-series variations in the value of variables that potentially affect the timing of turnover: (a) the annual average of the variables, and (b) the cold-season value of the variables. All variables are calculated based on their annual averages during the simulation period, with red showing values above or blue showing values below that average. The simulation results at the monitoring station were used for the analysis of water density and POC concentration. Water density is shown by the difference between the surface and bottom, and POC concentration is the average concentration of the surface and bottom. Other variable values are available from the model inputs.

Supplementary Figure S7

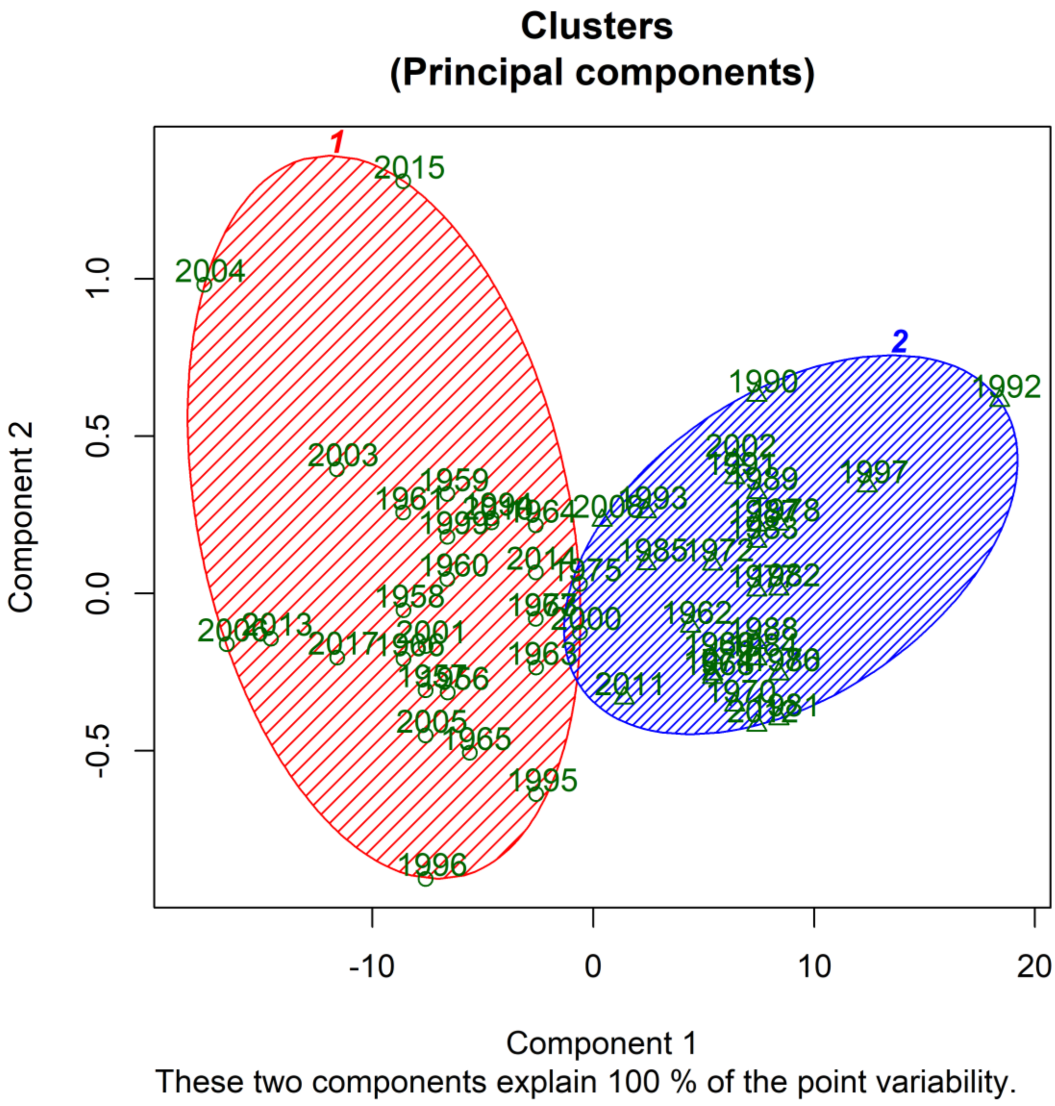

Figure S7. The plot of the cluster results (k-means, 2 clusters) using data for turnover timing and cold season air temperature. Generally, the relationship between the turnover timing and the cold season air temperature is different throughout 1976 to 1990 than it is during the rest period.

Supplementary Figure S8

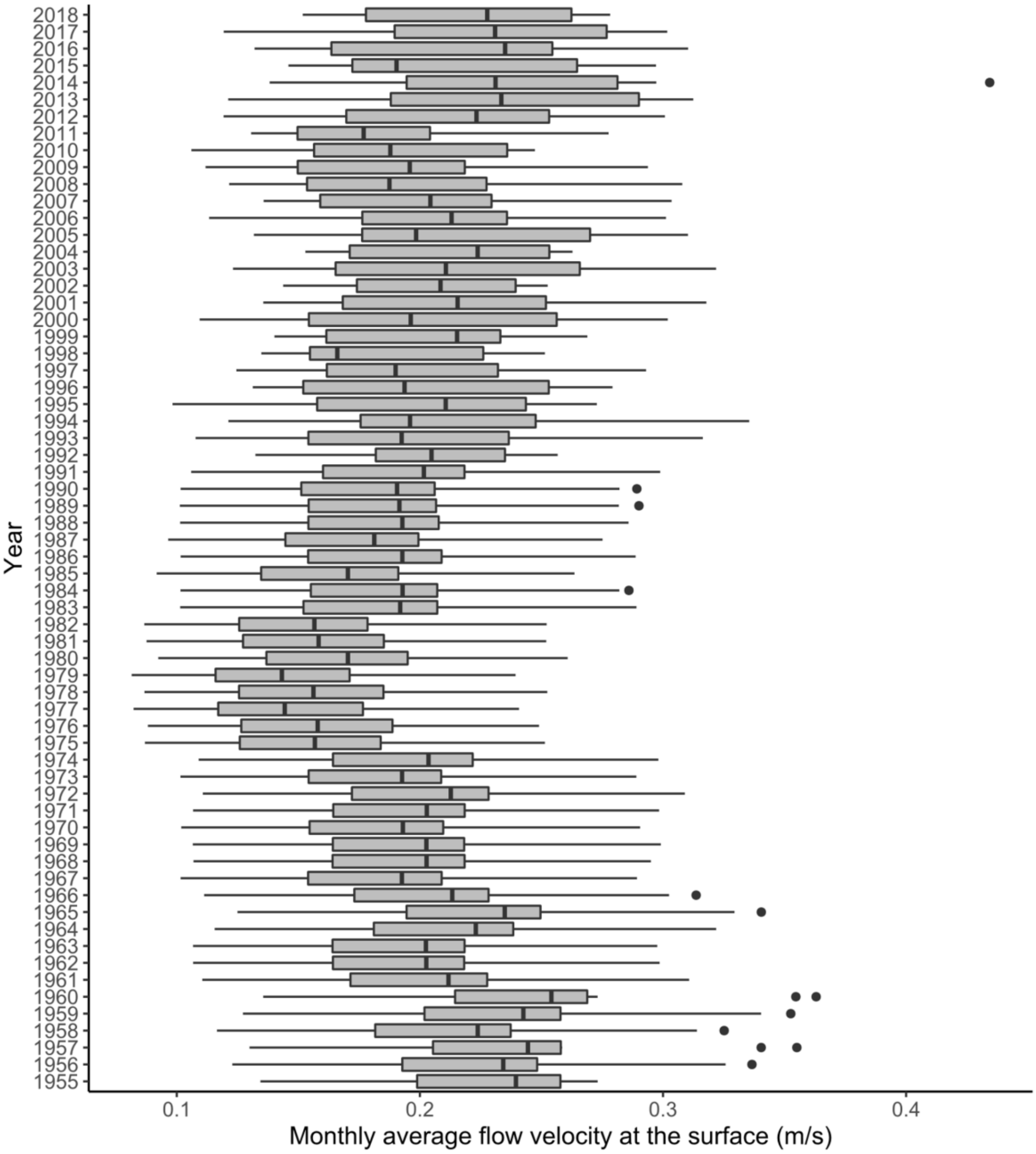

Figure S8. The monthly average of surface flow velocity over the simulation period. The simulation results at the monitoring station were used for the analysis.

Supplementary Figure S9

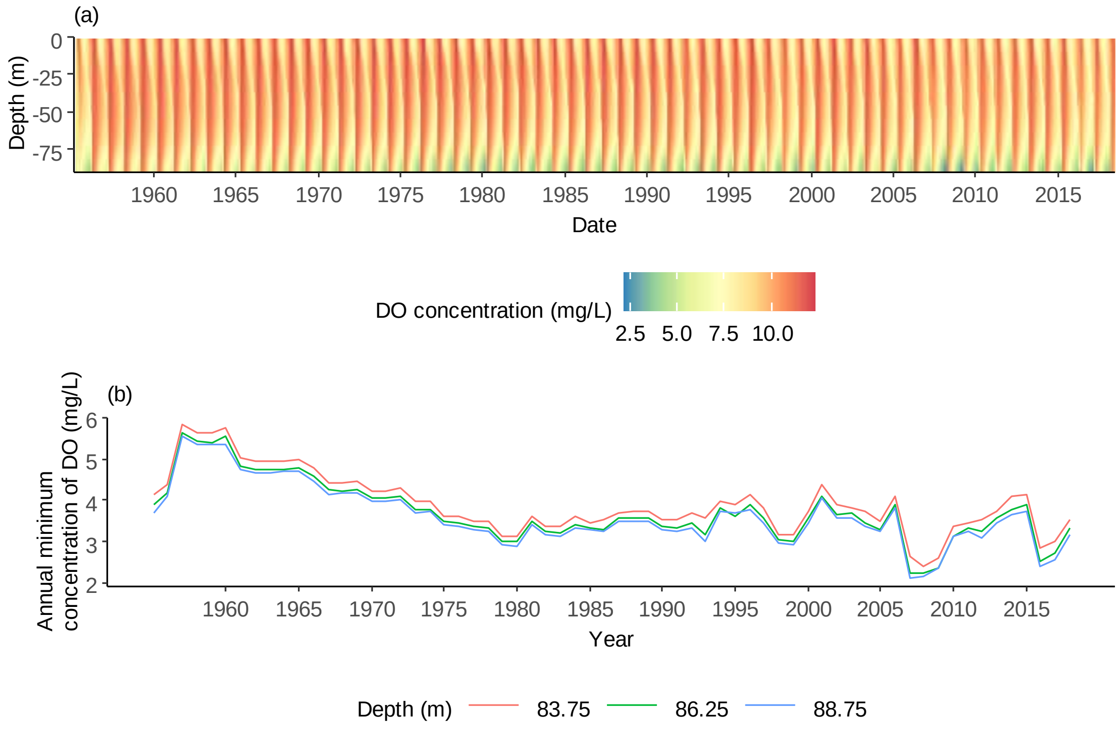

Figure S9. The variation in DO concentration at the monitoring station across all depths over the simulation period: (a) raw data shown in a contour plot and (b) annual minimum value for three bottom depths.

Supplementary Figure S10

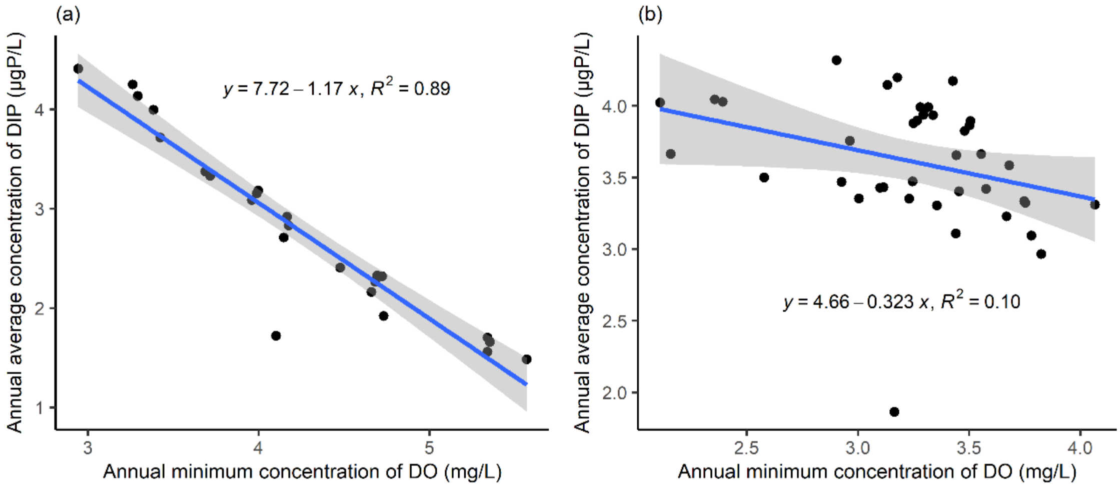

Figure S10. The relationship between the annual minimum concentrations of DO and annual average concentrations of DIP in Lake Biwa is shown in two different periods: (a) before 1980 when eutrophication was progressing, and (b) after 1980 when climate change became the dominant stressor.

Supplementary Figure S11

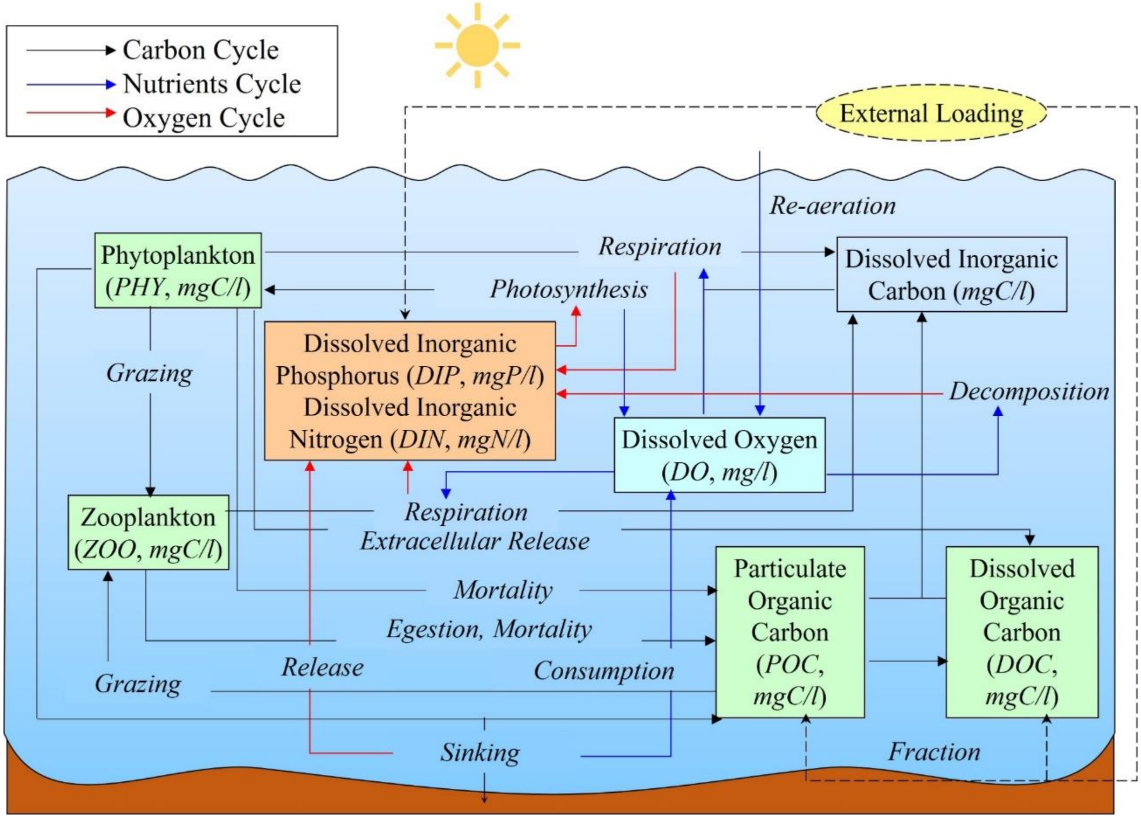

Figure S11. The conceptual structure of the pelagic ecosystem submodel. Phytoplankton (PHY), zooplankton (ZOO), dissolved inorganic phosphorus (DIP), dissolved inorganic nitrogen (DIN), dissolved oxygen (DO), particulate organic carbon (POC), and dissolved organic carbon (DOC) are the state variables.

Supplementary Figure S12

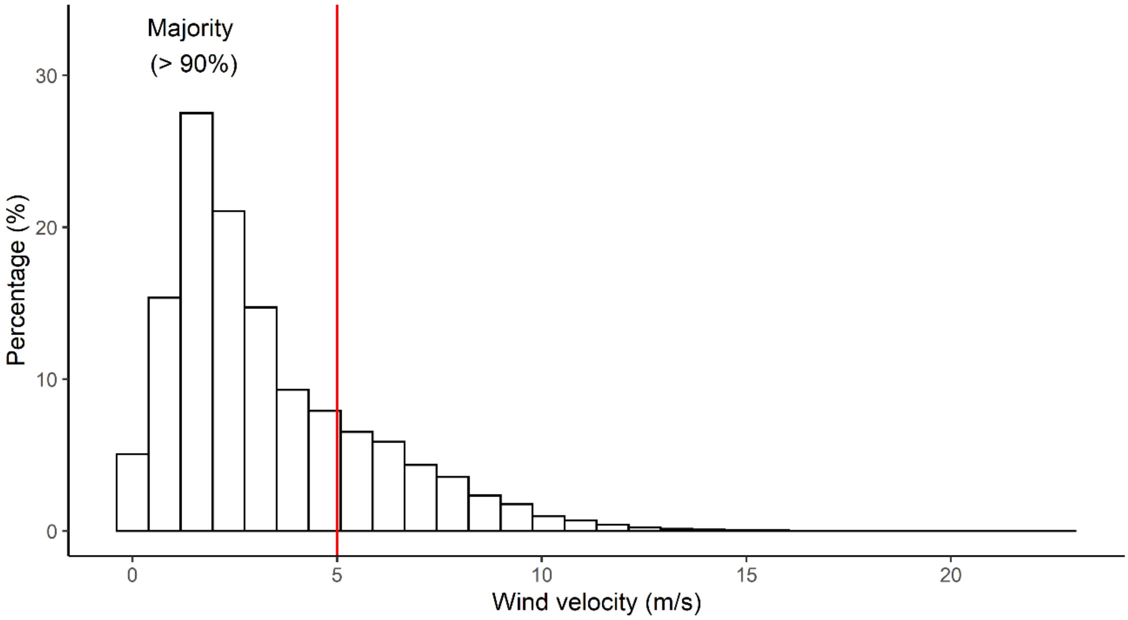

Figure S12. The density plot of wind velocity over the past 60 years in Lake Biwa. The majority (> 90%) of wind speed is slower than 5 m/s. Due to the slow wind speed, the piston velocity (K1, m/s) is constant (Gelda et al., 1996), and thus, is treated as the reaeration coefficient (K2, 1/s) in the simulation by dividing the unit volume of water (Kitazawa and Kumagai, 2007; Kawaguchi and Kojiri, 2010).

$$J = K1\ (Os - O)$$
$$K2 = K1/H$$

where J (mg/m^2/s) is oxygen flux; Os (mg/m^3) is saturation concentration of the dissolved oxygen; O (mg/m^3) is oxygen concentration in the water; H (m) is the water depth.

Reference

Gelda, R. K., Auer, M. T., Effler, S. W., Chapra, S. C., & Storey, M. L. (1996). Determination of reaeration coefficients: whole-lake approach. Journal of Environmental Engineering, 122(4), 269-275.

Kawaguchi, T. & Kojiri, T. (2010). Numerical experiments using the CASM to predict a regime shift in Lake Biwa. Annual Journal of Hydraulic Engineering, 54, 1465-1470.

Kitazawa, D. & Kumagai, M. (2007). A Numerical Study on Ecosystem in Lake Biwa by Hydrodynamic-Ecosystem Coupled Model (in Japanese with English abstract). Seisan kenkyu 59, 21–26. https://doi.org/10.11188/seisankenkyu.59.21
